# 

**DOI:** 10.1192/bjb.2025.3

**Published:** 2025-10

**Authors:** Claire Hilton

**Affiliations:** Centre for Interdisciplinary Research on Mental Health, Birkbeck University of London, London, UK. Email: claire.hilton6@gmail.com

**Figure:**
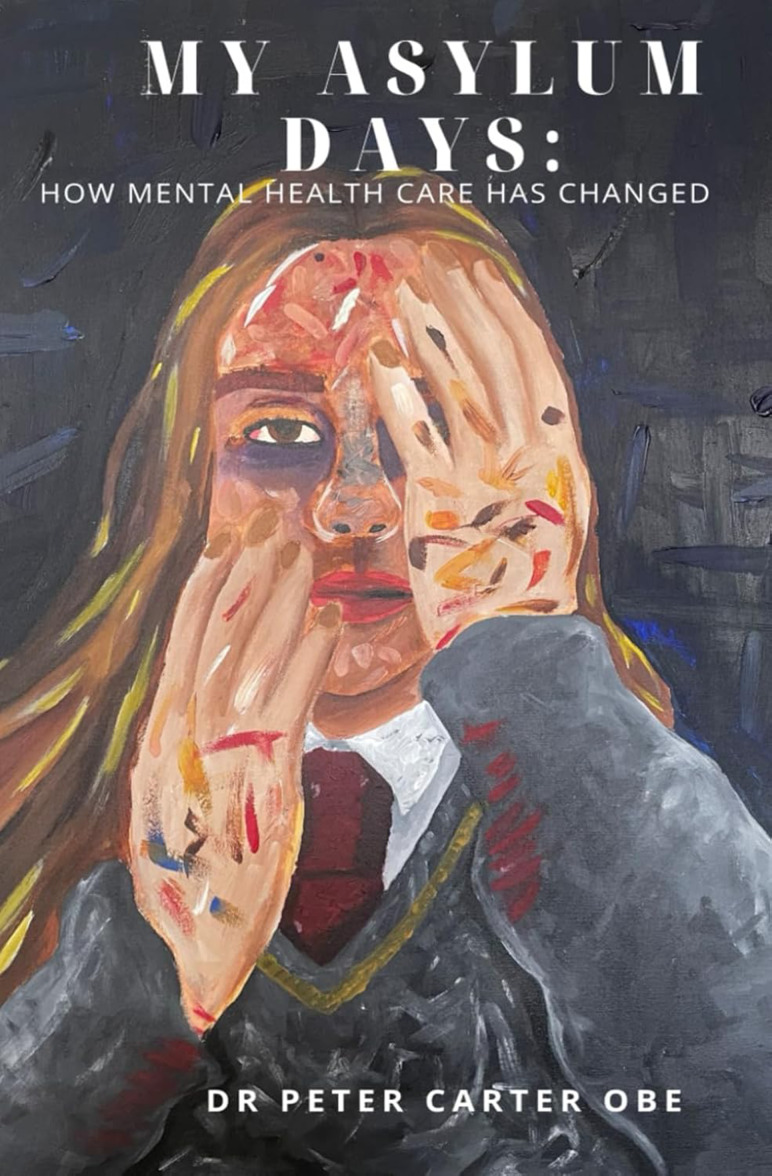

*My Asylum Days* reveals some of Peter Carter's encounters with patients, front-line staff and more senior people during his long National Health Service (NHS) psychiatric nursing and healthcare leadership career. The book is deeply reflective about mental health service policy and practice and how we treat our patients and colleagues. The story is about him, but it is also about us and where we stand today.

Dr Carter describes patterns of harmful leadership which he experienced early in his psychiatric nurse training in a Hertfordshire mental hospital in the late 1960s and which appear little different from those that occur today. At both times, usually well-intentioned staff could become overwhelmed by workloads and rules and regulations, affecting care for patients. Then, staff had ‘very little in terms of support. There was a macho culture and you were expected to just get on with life’. The latest NHS Staff Survey results^a^ are hardly reassuring that the work culture is significantly better today.

The author describes his career-long adventures of challenging the status quo, standing up for patients and fellow staff, and aiming to right wrongs and ‘confront issues without being confrontational’, even though whistleblowing was, and is, a scary process. He asked the magic word ‘why?’ about policy and practices, especially when they seemed substandard. We often fail to ask ‘why?’ and are perhaps too inclined to follow established structures, whether clinical or administrative. In this way, practices become normalised and perpetuate, even if they are far from ideal. He reflected that the multiple in-patient psychiatric hospital inquiries of the 1970s and 1980s ushered in a new era: of community inquiries. Too often, inquiry findings were accompanied by a rhetoric of intentions ‘to learn from this tragic event’, but recommendations were far from being implemented, especially those with resource implications.

This clearly written and passionate book has important messages for anyone working in mental health services: learn from your patients; listen to your juniors; be empathic and non-judgemental; and put human relationships at the heart of your clinical, leadership and policy work. *My Asylum Days* is a personal, thought-provoking and positive call to arms, to make us think about our roles and how we might shape future policy and practices in mental healthcare. In the words attributed to St Augustine of Hippo (354–430 CE): ‘Hope has two beautiful daughters: their names are anger and courage. Anger at the way things are, and courage to see that they do not remain the way they are’. Peter Carter's story demonstrates all three.

## Footnotes

a NHS Survey Coordination Centre. *NHS Staff Survey 2023*. National Results Briefing, 2024 (https://www.nhsstaffsurveys.com/results/national-results/).

